# The P2X7R-antagonist AFC-5128 ameliorates chronic experimental autoimmune encephalomyelitis in a preventive and therapeutic paradigm

**DOI:** 10.3389/fimmu.2025.1554999

**Published:** 2025-04-16

**Authors:** Robert Hoffrogge, Anna Karachunskaya, Neele Heitmann, Steffen Haupeltshofer, Xiomara Pedreiturria, Katharina Klöster, Verian Bader, Konstanze F. Winklhofer, Michael Hamacher, Bert Klebl, Ralf Gold, Klaus Dinkel, Ingo Kleiter, Simon Faissner

**Affiliations:** ^1^ Department of Neurology, St. Josef-Hospital, Bochum, Germany; ^2^ Translational Neurology Group, Department of Clinical Science, Lund University, Lund, Sweden; ^3^ Molecular Cell Biology, Ruhr-University, Bochum, Germany; ^4^ Affectis Pharmaceuticals AG, Dortmund, Germany; ^5^ KHAN Technology Transfer Fund I GmbH & Co KG, Dortmund, Germany; ^6^ Lead Discovery Center GmbH, Dortmund, Germany; ^7^ Behandlungszentrum Kempfenhausen für Multiple Sklerose Kranke gemeinnützige GmbH, Berg, Germany

**Keywords:** P2X7R, progressive multiple sclerosis, microglia, neuroinflammation, neuroprotection

## Abstract

**Background:**

Multiple sclerosis (MS) is characterized by chronic inflammation driven by central nervous system (CNS)-resident immune cells such as microglia, especially during the progressive phase of the disease. The P2X7 receptor (P2X7R), a risk protein for MS, is ubiquitously expressed on immune cells. AFC-5128, a CNS-penetrating small molecule inhibitor of P2X7R, is a promising agent for the treatment of autoimmune diseases such as MS.

**Methods:**

*In vitro*, the effects on the calcium influx of primary murine microglia were assessed via Fluo-4 calcium imaging. *In vivo*, MOG_35-55_ immunized C57BL/6 mice were treated with AFC-5128, fingolimod (FTY) or vehicle in different treatment paradigms. The mice were scored daily. Microglial marker expression, immune cell phenotyping and serum cytokine analyses were performed via flow cytometry. Immune cell infiltration, demyelination and Iba1^+^/CD3^+^ cells were detected in spinal cord cross-sections. The effects of MOG_35-55_ T-cell restimulation were assessed *in vitro*.

**Results:**

*In vitro*, treatment of primary microglia with 10 µM AFC-5128 reduced the influx of calcium following ATP stimulation (p<0.0001). *In vivo*, treatment of mice with AFC-5128 led to a reduction in overall EAE scores in acute and chronic EAE, with the best effects using 200 mg/kg body weight AFC-5128 (p<0.0001). Peripheral immune cell subsets (B cells, T cells and macrophages) and serum cytokine levels of chronic EAE mice treated in a therapeutic paradigm were not affected. While the expression of homeostasis markers of microglia in AFC-5128-treated mice was not affected, there was a trend toward lower expression of phagocytosis-associated markers. Late therapeutic treatment with AFC-5128 had only mild effects on chronic EAE.

**Conclusion:**

The treatment of EAE mice with AFC-5128 improved acute and chronic EAE in different treatment paradigms, with positive effects on histological markers and slight modulation of microglial marker expression. Mechanistically, calcium influx of microglia was reduced following AFC-5128 treatment, which implies the ability of AFC-5128 to stabilize calcium homeostasis. Therefore, therapeutic inhibition of P2X7R via AFC-5128 has the potential for translation into a treatment of both relapsing and progressive forms of multiple sclerosis.

## Introduction

Multiple sclerosis (MS) is a chronic inflammatory disease of the central nervous system (CNS) that is characterized by neurodegeneration in the brain and spinal cord with increasing disability (1, 2). Most patients initially present with relapsing-remitting disease (RRMS), which is characterized by the migration of MOG-reactive T lymphocytes and the activation of B cells, leading to damage of the myelin sheaths and neurological symptoms. After 15–20 years of disease, the risk of transition into a secondary progressive course (SPMS), characterized by a slow progressive accumulation of disability with new relapses occurring more rarely, increases (3). Approximately 10–15% of patients with MS are initially assigned to a primary-progressive type of disease (PPMS) (1). In the last few decades, a wide range of disease-modifying therapies (DMTs) have been developed and established for the treatment of RRMS, whereas there is still a lack of effective therapies for progressive forms (4, 5). The reasons for this might be the differing pathomechanisms of progressive MS, which cannot be treated effectively with existing DMTs (2, 6).

Progressive MS is characterized by chronic inflammation, which is caused mainly by diffuse modulation of resident glia behind a relatively closed blood-brain barrier (7). Microglial nodules can be found more frequently in normal-appearing white matter in patients with progressive disease (8). The purinergic P2X7 receptor (P2X7R), a ligand-gated ion channel, is becoming increasingly interesting in various autoimmune diseases, including multiple sclerosis (9–11). It promotes morphological changes and the proliferation of microglia (9, 12). Different studies have revealed its involvement in various aspects of MS. For example, the receptor is significantly increased in oligodendrocyte-derived extracellular vesicles from the serum of PPMS patients (13), and its expression is elevated on reactive astrocytes and microglia in MS lesions (14, 15), as well as in the normal-appearing axonal tracts of MS patients (16). *In vivo*, sustained activation of P2X7R causes lesions that are reminiscent of MS lesions (16), and *in vitro*, P2X7R is associated with an inflammation-associated phenotype of human microglia (17). Rare coding variants of the P2X7R may play a role in the pathogenesis and modulation of MS (18, 19). Despite the growing interest in the role of the P2X7 receptor in MS, there are significant gaps in our understanding of the therapeutic potential of its inhibition.

Here, we investigated the effects of the P2X7R inhibitor AFC-5128, a CNS-penetrating small molecule, in an established animal model of chronic MS, experimental autoimmune encephalomyelitis (EAE). Our objective was to determine whether inhibiting P2X7R can modulate disease progression and microglia to provide new insights into the therapeutic potential of targeting P2X7R in MS.

## Methods

### Primary microglia

Cortices from P0–2 C57BL/6 mouse pups were manually obtained and microglia were prepared for experimental analysis as previously described (20). A total of 1.5x10^5^ cells in 200 µl of complete DMEM/F12 GlutaMax™ medium (**Supplementary Table S2a**) were seeded into 0.8 mm high channel slides (Ibidi). After 1 h, the medium was changed to complete Neurobasal B27 medium (**Supplementary Table S2b**) to ensure a pure microglial population.

### Fluo-4 calcium imaging

After 24 hours of cultivation and adaptation to the environment, the cells were washed and loaded with Fluo-4 AM solution (**Supplementary Table S2c**), followed by incubation in the dark for 30 minutes at 37°C and 15 minutes at RT. Thereafter, the cells were treated with 10 µM AFC-5128 (Affectis) in HBSS w/o Ca^2+^ for 15 minutes or received DMSO at the appropriate dilution as a control. The channel slides were directly connected to a peristaltic pump to acquire a fluorescence intensity baseline with 1–2 ml/min 20 mM glucose in PBS via a Zeiss Elyra^®^ PS.1 microscope (Carl Zeiss, Germany). The mean intensity data were acquired by defining regions of interest (ROIs) in each visible cell. Next, stimulation solution (**Supplementary Table S2d**) was added to the system, followed directly by the addition of the baseline solution to avoid air pockets. The mean intensity was measured with a frequency of one picture/sec until the baseline level was reached again. The control cells received the stimulation solution with DMSO. The acquired mean intensity data were analyzed and processed with Zen Blue (version 3.5, Carl Zeiss Microscopy, Oberkochen, Germany), whereby the measured mean fluorescence intensity of each individual cell was normalized to the individual average baseline intensity.

### Experimental autoimmune encephalomyelitis

All animal experiments were approved by the animal care committee of North Rhine-Westphalia, Germany (LANUV, no. 81-02.04.2021.A368).

The mice were kept under environmentally controlled open caging conditions with a constant temperature and a 12:12 h dark–light cycle. The mice had access to food and water *ad libitum*. Prior to the start of the experiment, the mice were allowed to adapt to the environment and handling conditions for at least two weeks.

For all experiments, 8- to 10-week-old C57BL/6J mice (Charles River, Sulzfeld, Germany) were immunized with 100 µg of myelin oligodendrocyte glycoprotein 35-55 peptide (MOG_35-55_, Peptide 2, Genosphere Biotech) in complete Freund’s adjuvant (CFA) by subcutaneous injection into both hind flanks. On days 0 and 2 post immunization, 250 ng of pertussis toxin (PTX, Sigma Aldrich) dissolved in PBS was injected intraperitoneally to induce blood–brain barrier leakage as previously described (21). The mice were weighed and scored daily for clinical signs according to a previously defined 10-point scale: 0: no signs of disability; 1: tail paresis; 2: complete tail paralysis; 3: missing compensatory movements while walking; 4: ataxia; 5: moderate hind leg paresis; 6: complete paresis of one hind leg or stronger paresis of both hind legs; 7: paraplegia; 8: tetraparesis; 9: moribund; 10: death (21, 22). Animals with a score of 7 and/or loss of >20% of body weight were euthanized according to local animal care committee guidelines.

Before the start of treatment, the mice were randomized according to weight in the prophylactic experimental group or according to the score and weight in the therapeutic experiments. The mice were treated in a blinded manner with AFC-5128 at different dosages (100, 200 and 400 mg/kg body weight), 1 mg/kg body weight fingolimod (Sigma Aldrich) or vehicle from 1 dpi (prophylactic paradigm), after the peak of disease (therapeutic paradigm) or during the chronic phase (late therapeutic) by oral gavage. AFC-5128 (Affectis) was dissolved in a 50/50 mixture of carboxymethylcellulose and rapeseed oil. Fingolimod was dissolved in 100% rapeseed oil.

At the termination of the experiments, blood samples were collected via cardiac puncture for serum cytokine level determination and immune cell isolation for flow cytometry. Microglia from the brain, lymph node cells and splenocytes were prepared for flow cytometry analysis. The spinal cord segments were fixed and embedded in paraffin for histological analysis.

For *in vitro* restimulation of T cells, female C57BL/6 mice were immunized with MOG_35-55_ and treated with AFC-5128 or vehicle or left untreated (control). After 10 days, the lymph node-derived naïve T cells were isolated and incubated (basic medium: RPMI-1640, 10% FCS, 2 mM L-glutamine, P/S, 50 μM β-mercaptoethanol) with vehicle control, AFC 100 mg/kg or 200 mg/kg. After preconditioning, the T cells were labeled with CFSE. In parallel, BMDCs were isolated and differentiated into dendritic cells (basic medium, 20 ng/mL GM-CSF, 10 ng/mL IL-4). BMDCs were restimulated with 1 or 10 µg/ml MOG_35-55_ peptide for 24 h to achieve effective antigen processing. Preconditioned T cells (2.25x10^6^) were incubated with 2.5x10^5^ BMDCs in basic medium. Anti-CD3/28 antibody stimulation served as a positive control, whereas unstimulated condition was used as a negative control. AFC-5128 was used at plasma-equivalent dosages of 5 µg/ml for 100 mg/kg and 10 µg/ml for 200 mg/kg. After 4 days of incubation, T-cell proliferation was analyzed via flow cytometry.

### Flow cytometry

We present the cell populations as frequency of a parent population in the results section but show the absolute cell counts of the subpopulations in **Supplementary Figures S2**, **S3**. The absolute cell counts were calculated by multiplication of the frequency of living cells by the total number of isolated cells per brain, blood, spleen or inguinal lymph nodes sample of each mouse.

### Microglia

To analyze the different subtypes of microglia, the brains were extracted and kept in ice-cold complete RPMI medium (**Supplementary Table S1a**). Microglia were dissociated as previously described (23), extracted via Percoll™ (24), subsequently stained with antibodies (**Supplementary Table S1b**) and analyzed via flow cytometry using a BD FACS Celesta™ (BD Biosciences). Data were obtained via FACSDiva Software (BD Biosciences) and analyzed via FlowJo (Becton Dickinson & Company, version 10.0.7). CD11b^+^ CD45^int^ microglia were identified after the exclusion of doublets and dead cells (**Supplementary Figure S1A**).

### Immune cells

Cells from the blood, spleen and lymph nodes were isolated as previously described (21). The cells were stained with fluorophore-coupled antibodies (**Supplementary Table S1b**) and analyzed via flow cytometry using a BD FACS Celesta™ (BD Biosciences). Data were obtained via FACSDiva (BD Biosciences) and analyzed via FlowJo (Becton Dickinson & Company, version 10.0.7). CD45^+^ lymphocytes and monocytes were identified after the exclusion of doublets and dead cells. Subpopulations are given as a percentage of the related parent.

### Histology

Paraffine sections were deparaffinized with Roti-Histol (Carl Roth) and rehydrated with a descending ethanol series prior to being stained with Mayer′s hemalum solution (Sigma-Aldrich) and 0.5% eosin solution (Carl Roth) to investigate cell infiltration and Luxol Fast Blue (Thermo Scientific Chemicals) with a periodic acid Schiff reaction (Schiff’s reagent, Carl Roth) to demarcate demyelinated areas. For representative immunohistochemistry, after heat-mediated antigen retrieval with 10 mM citrate buffer and blocking with normal goat serum (Biozol), the sections were stained with anti-Iba1 and costained with anti-CD3 and secondary antibodies (**Supplementary Table S1c**) to investigate microglia/macrophages and infiltrating T cells. The cell nuclei were stained with DAPI-Fluoromount. Pictures were acquired with an Axio Observer^®^ microscope (Carl Zeiss, Germany) using Zen Blue (version 3.5, Carl Zeiss Microscopy, Oberkochen, Germany). All the images were merged, blinded and evaluated via ImageJ (Wayne Rasband and contributors, National Institutes of Health, USA, version 1.53k). HE- and LFB-stained samples were evaluated with manual definitions of infiltrates and demyelinated areas. Representative Iba1-CD3 staining was evaluated by counting double-positive DAPI-Iba1- and DAPI-CD3-positive cells. The gray matter was excluded from the analysis.

### LEGENDplex™ Mouse Inflammation Panel predefined kit for serum cytokine analysis

Cytokines in the serum supernatants were analyzed via the LEGENDplex™ Mouse Inflammation Panel Kit (BioLegend). All samples were analyzed in duplicates via flow cytometry using a BD FACS Celesta™ (BD Biosciences) and FACSDiva (Becton Dickinson & Company). The acquired data were analyzed via LEGENDplex™ Data Analysis Software (BioLegend).

### Statistical analysis

The statistical analysis was performed via Prism 10.1.1 software (GraphPad Software LLC, Boston, MA, USA). Nonparametric data were analyzed via the Kruskal–Wallis test with *post hoc* analysis via Dunn`s multiple comparisons test, as shown in the respective figure legends. Correlations were analyzed via nonparametric Spearman correlation. The values of the control and pretreated cells in the calcium imaging experiments were analyzed via the Mann–Whitney test. The data are shown as the means ± standard errors of the means (SEMs). A p-value <0.05 was considered to indicate statistical significance. Significances are depicted as * p<0.05; ** p<0.01; *** p<0.001 and **** p<0.0001.

## Results

### AFC-5128 reduces ATP-mediated Ca^2+^-uptake by primary murine microglia *in vitro*

The P2X7 receptor is a ubiquitously expressed membrane-bound ligand-gated ion channel that is primarily selective for small cations following the binding of extracellular ATP (11, 25). In the presence of high and long-lasting ATP concentrations, receptor activation can result in pore formation of the receptor, which allows the passage of large cations, leading to cell death (25–27).

**Figure 1:**
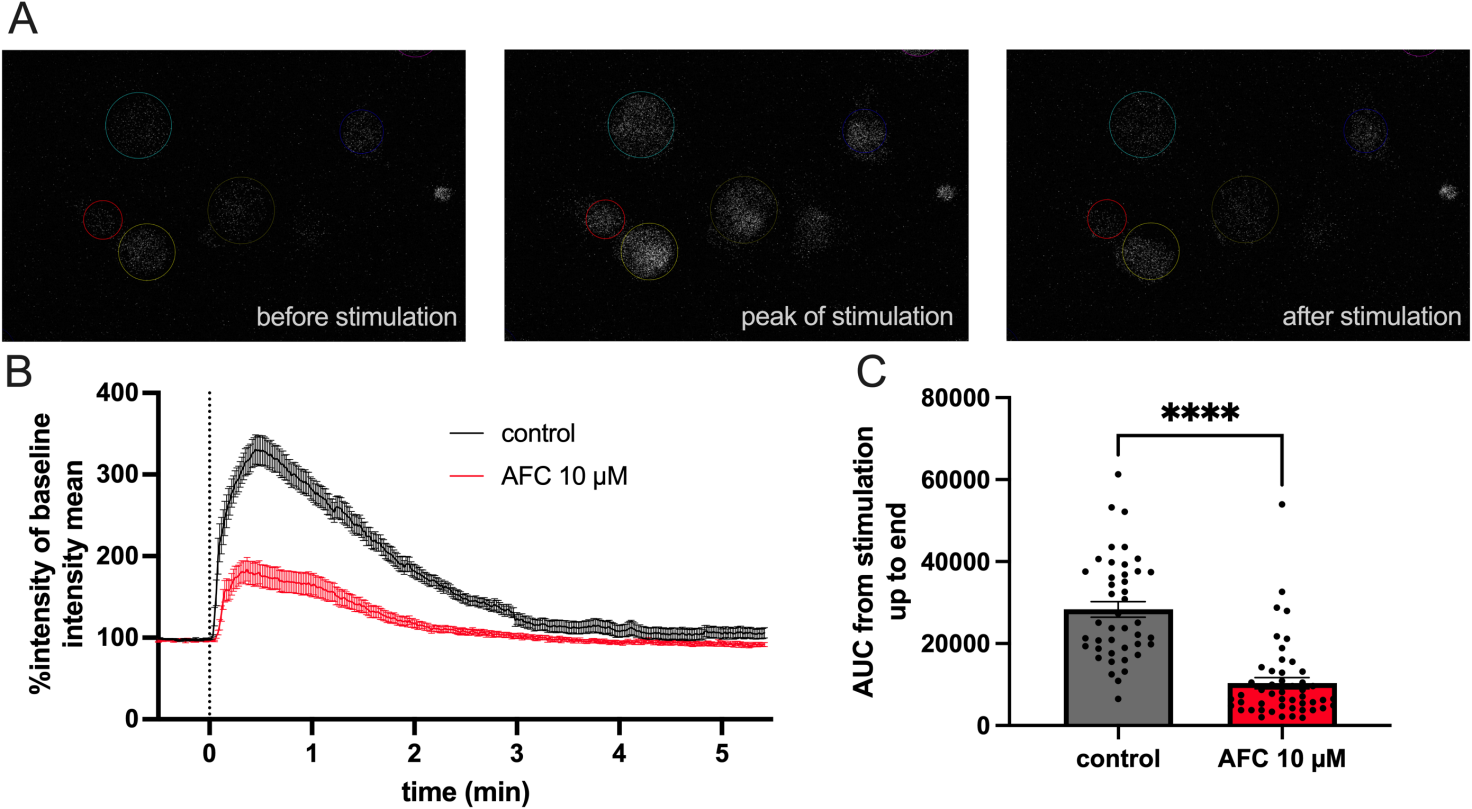
AFC-5128 reduces ATP-gated calcium uptake in primary murine microglia. **(A)** Example fluorescence images of cultured primary murine microglia loaded with Fluo-4 AM, showing Ca^2+^ influx and signaling before, during and after the application of 2.5 mM CaCl_2_ plus 5 mM ATP. **(B)** Averaged data from time-lapse imaging of dynamic fluorescence changes in microglia induced by CaCl_2_ and ATP stimulation (black curve) or with 10 µM AFC-5128 pretreatment and subsequent stimulation (red curve). Data from a total of 3 experiments with a total of 43 (control) and 49 (AFC-5128) cells were normalized to the average fluorescence intensity measured before stimulation in each cell. **(C)** Pretreatment with 10 µM AFC-5128 reduced Ca^2+^-uptake following ATP stimulation, as shown by the significantly lower AUC of the intensity graphs. The data are shown as the means ± SEMs. The data were analyzed via the Mann–Whitney test. Significances are depicted as **** p < 0.0001. CaCl_2_, calcium chloride; ATP, adenosine triphosphate; AUC, area under curve.

To understand the potential effects of AFC-5128 on microglial calcium homeostasis, we performed *in vitro* live-cell imaging experiments using primary murine microglia. First, we established a suitable effective stimulation using 2.5 mM CaCl_2_ with 5 mM ATP (not shown). Prior to stimulation, the cells were loaded with Fluo-4 AM, a small-molecule reagent whose reaction with cytosolic calcium results in an increase in the intracellular fluorescence intensity. Pretreatment with 10 µM AFC-5128 led to a significantly reduced fluorescence intensity after stimulation with 2.5 mM CaCl_2_ and 5 mM ATP (p<0.0001; **Figure 1**), which corresponds to a reduced influx of calcium. Hence, AFC-5128 can strongly regulate the function of P2X7R and thus the calcium homeostasis of microglia *in vitro*.

### AFC-5128 improves acute experimental autoimmune encephalomyelitis

**Figure 2:**
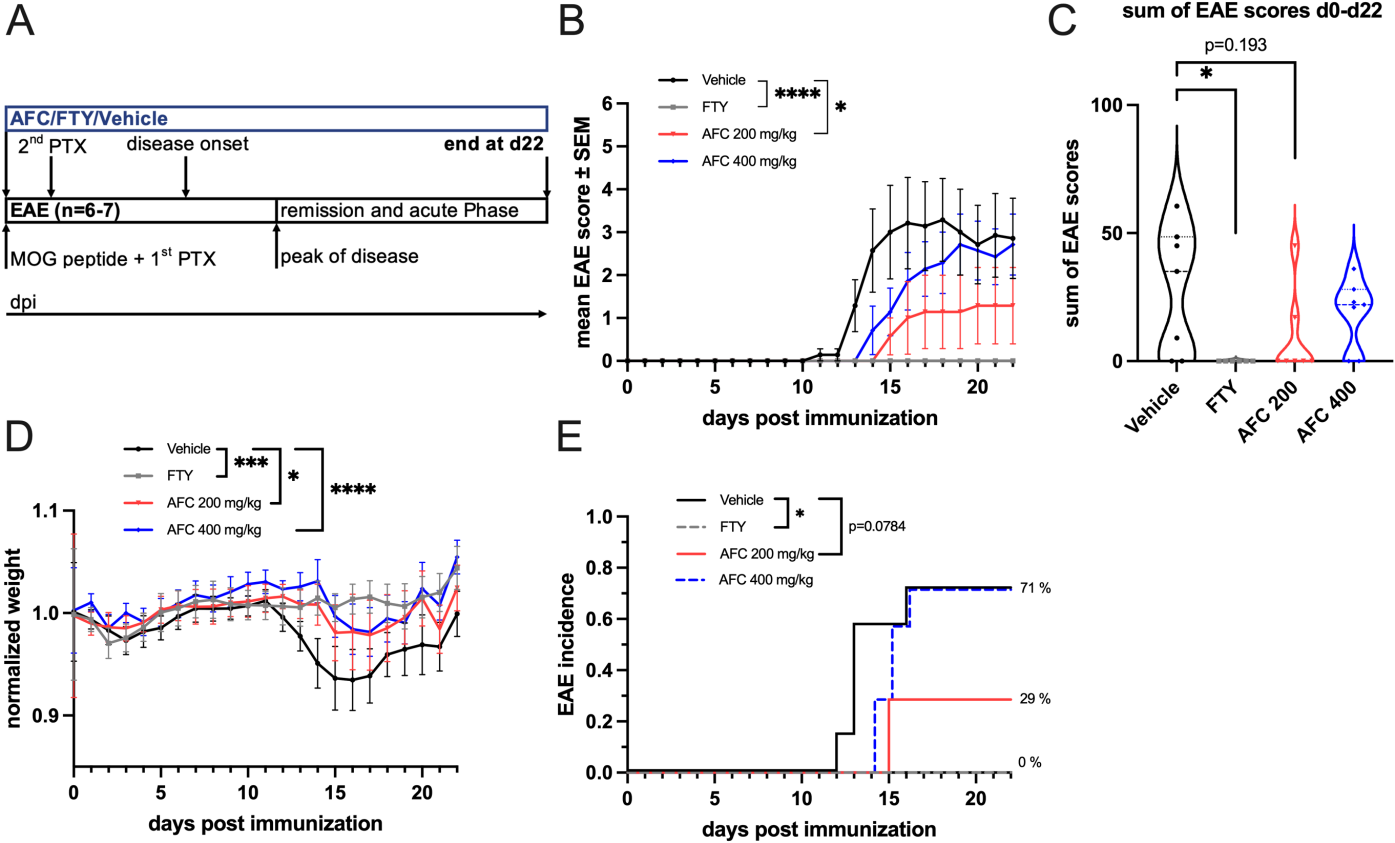
Prophylactic AFC-5128 treatment improves acute MOG-EAE in C57BL/6 mice. **(A)** 8- to 10-week-old C57BL/6 mice were immunized with s.c. MOG_35-55_ injection and treated i.p. with PTX on days 0 and 2 postimmunization. The mice were treated 2x/d with vehicle, 1 mg/kg body weight per day FTY or different dosages of AFC-5128 by oral gavage from day 0 to day 22. **(B)** Overall EAE scores were significantly reduced in the 200 mg/kg body weight AFC-5128 group than in the control group. 1 mg/kg FTY, as a positive control, completely suppressed clinical EAE symptoms. **(C)** The sum of the EAE scores, which reflects the disease severity, tended to decrease when 200 mg/kg AFC-5128 was used (p=0.193), reaching significance when FTY was used. **(D)** Weight loss was significantly lower in AFC-5128- and FTY-treated mice than in vehicle-treated mice. **(E)** FTY significantly reduced the incidence of the EAE (p<0.05). n=6–7 per group. The data are shown as the means ± SEMs. Weight was normalized to the respective group mean. EAE Score and weight data **(B, C, D)** were analyzed via the Kruskal-Wallis test with *post hoc* Dunn’s multiple comparisons analysis. The incidence data **(E)** were analyzed with log-rank test. Significances are depicted as *p < 0.05; ***p< 0.001; **** p < 0.0001. EAE, experimental autoimmune encephalomyelitis; MOG, myelin oligodendrocyte glycoprotein; s.c., subcutaneously; i.p., intraperitoneally; PTX, pertussis toxin; FTY, fingolimod.

To investigate the effects of AFC-5128 in acute MOG-EAE, we treated C57BL/6 mice in a prophylactic paradigm with different dosages of AFC-5128 applied by oral gavage from the day of MOG-immunization and throughout the entire experimental period (**Figure 2A**). Mice were treated with 1 mg/kg body weight FTY per day as a positive control because of its known strong effect on EAE (28, 29). As expected, compared to the control treatment, FTY treatment led to complete suppression of clinical signs (**Figure 2E**) and thus significantly reduced EAE scores (p<0.0001; **Figure 2B**), the sum of EAE scores (p<0.05; **Figure 2C**), weight loss (p<0.001; **Figure 2D**) and incidence (p<0.05; **Figure 2E**). AFC-5128 at a dosage of 200 mg/kg body weight led to an overall reduction in EAE scores (p<0.05; **Figure 2B**). However, the disease severity assessed by the sum of the EAE scores did not differ (p=0.193; **Figure 2C**). The overall weight loss as a marker for general health was significantly reduced in 200 mg/kg AFC-5128-treated mice (p<0.05; **Figure 1D**), as well as in 400 mg/kg AFC-5128-treated mice (p<0.0001; **Figure 2D**). In the vehicle-treated mice, the median day of onset of EAE was day 13, compared to 15 in 400 mg/kg AFC-5128 treated mice. FTY-treated mice did not develop EAE. Treatment with 200 mg/kg AFC resulted in an EAE incidence of less than 50% (29%, **Figure 2E**). However, this effect was borderline nonsignificant compared to vehicle (p=0.0784, **Figure 2E**).

### Therapeutic administration of AFC-5128 ameliorates chronic EAE

**Figure 3:**
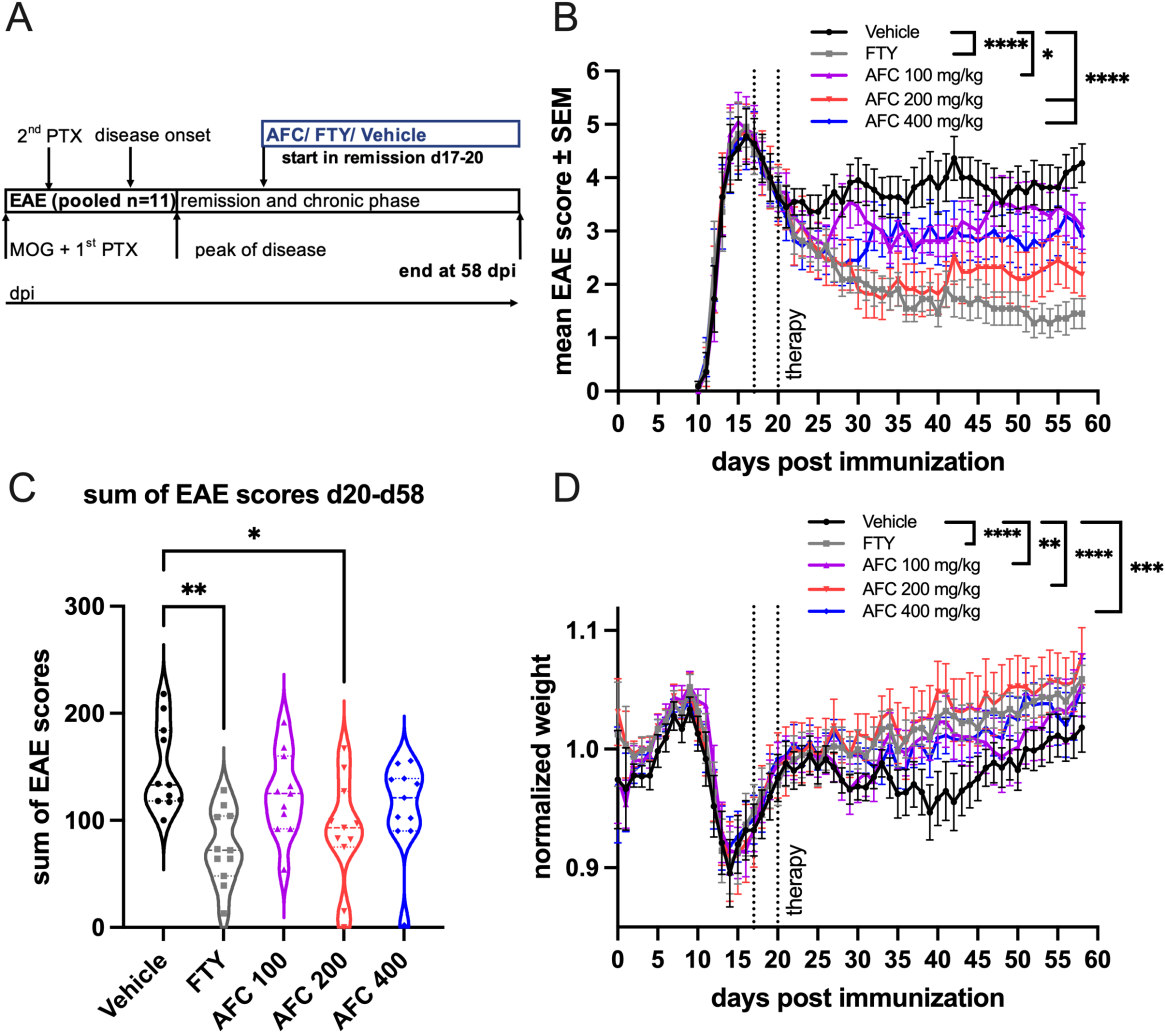
Therapeutic AFC-5128 treatment ameliorates chronic MOG-EAE in C57BL/6 mice. **(A)** 8- to 10-week-old C57BL/6 mice were immunized with s.c. MOG_35-55_ injection and treated i.p. with PTX on days 0 and 2 post immunization. The mice were treated 2x/d with vehicle, 1 mg/kg body weight FTY per day or different dosages of AFC-5128 by oral gavage from days 17- -20 (1^st^ exp. d17; 2^nd^ exp. d20; start in remission) until day 58 postimmunization. **(B)** Treatment with AFC-5128 reduced overall EAE scores. FTY, as a positive control, was effective as well. **(C)** The treatment effects are reflected in a significantly reduced disease severity, as assessed by the sum of EAE scores, when FTY and 200 mg/kg AFC-5128 were used. **(D)** Weight loss was significantly reduced in AFC-5128- and FTY-treated mice than in vehicle-treated mice. Data from two independent experiments with the same settings were pooled (1^st^ exp. n=5, 2^nd^ exp. n=6, total n=11 per group). The data are shown as the means ± SEMs. Weight was normalized to the respective group mean. The data were analyzed using the Kruskal-Wallis test with *post hoc* Dunn’s multiple comparisons analysis. Significances are depicted as *p < 0.05; **p < 0.01; *** p < 0.001; **** p < 0.0001. EAE, experimental autoimmune encephalomyelitis; MOG, myelin oligodendrocyte glycoprotein; s.c., subcutaneously; i.p., intraperitoneally; PTX, pertussis toxin; FTY, fingolimod.

After AFC-5128 showed positive effects on acute EAE, we wanted to evaluate whether it also has a positive effect on chronic MOG-EAE if applied in a therapeutic treatment paradigm. Treatment initiation was performed after the peak of disease, at which time most of the mice were in remission (**Figure 3A**), and three different dosages of AFC-5128 were used, as well as 1 mg/kg FTY per day as a positive control. The clinical data of two independent experiments with the same settings were pooled together (1^st^ exp. n=5, 2^nd^ exp. n=6, total n=11 per group). Treatment with AFC-5128 significantly reduced the overall EAE scores, with the best effects observed with 200 and 400 mg/kg AFC-5128 (both p<0.0001; **Figure 3B**). The lowest dose of 100 mg/kg AFC-5128 significantly reduced the overall EAE scores compared with that of the vehicle group (p<0.05; **Figure 3B**). Additionally, the overall weight loss was significantly reduced in all the AFC-5128-treated groups, with least weight loss occurring in the 200 mg/kg AFC-5128 group (p<0.0001; **Figure 3D**). As in the acute EAE setting, FTY showed a significant reduction of the overall EAE scores and weight loss compared with the control group (p<0.0001; **Figures 3B, D**). However, the disease severity, as assessed by the sum of the EAE scores, was significantly reduced only in the groups treated with FTY (p<0.01; **Figure 3C**) and 200 mg/kg AFC (p<0.05, **Figure 3C**).

### Therapeutic treatment with AFC-5128 reduces infiltration and demyelination in the spinal cord

**Figure 4:**
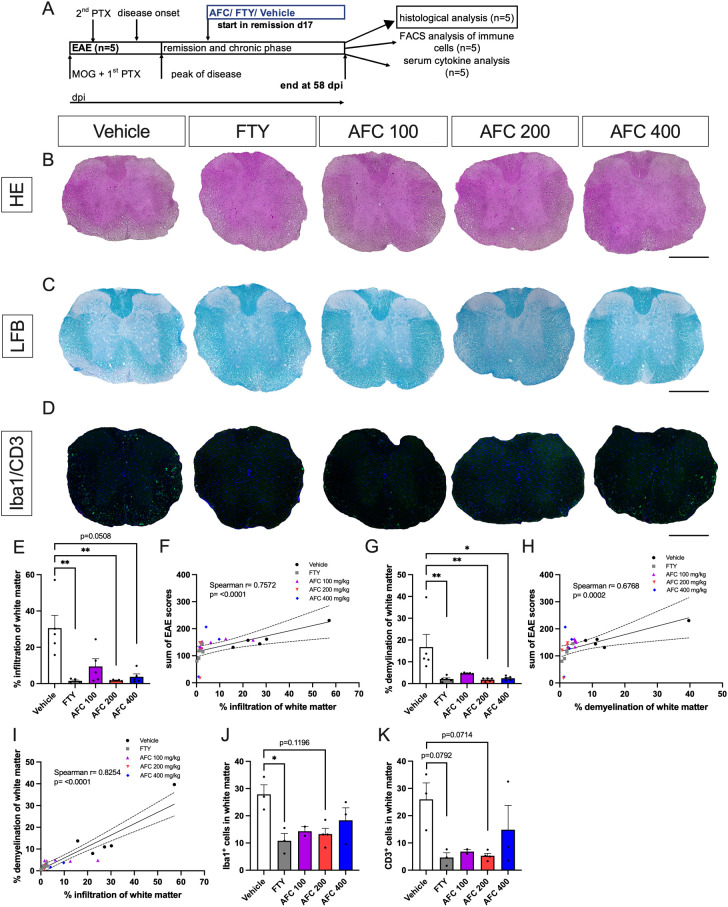
Therapeutic AFC-5128 administration reduces cell infiltration and demyelination of white matter in thoracic spinal cord sections from chronic EAE mice. Histological examination of mice from the first experiment with the therapeutic treatment approach was performed (n=5; **(A)**). The tissues were collected on day 58 after immunization **(A)**. Representative HE **(B)**, LFB **(C)** and Iba1^+^/CD3^+^ **(D)** images of thoracic spinal cord sections. **(E)** Both FTY and 200 mg/kg AFC-5128 led to significantly decreased infiltration in thoracic spinal cord sections of chronic EAE mice. **(F)** Greater cell infiltration and greater disease activity were correlated (Spearman r= 0.7572; p<0.0001). **(G)** Demyelination was also significantly reduced, with the strongest effects observed with FTY and 200 mg/kg AFC-5128. **(H)** Similar to infiltration, demyelination and the sum of EAE scores were correlated (Spearman r=0.6768; p= 0.0002). **(I)** Cell infiltration and demyelination of white matter were correlated as well (Spearman r= 0.8254; p<0.0001). A representative evaluation of subsets of the groups (n=2-4 per group) revealed a trend toward lower numbers of **(J)** Iba1^+^ and **(K)** CD3^+^ cells in the white matter of the AFC-5128- and FTY-treated mice, with a significant reduction in the number of Iba1^+^ cells in the FTY-treated group. For histological analysis of cell infiltration and demyelination (n=5 per group), thoracic spinal cord sections were analyzed in nine technical replicates for each mouse. For representative Iba1/CD3 analysis (n=2-4), sections were analyzed in six technical replicates for each mouse. The data are shown as the means ± SEMs. Infiltration, demyelination and fluorescence staining **(E, G, J, K)** were analyzed via the Kruskal–Wallis test with *post hoc* Dunn’s multiple comparisons analysis. Correlation analysis **(F, H, I)** was performed via Spearman correlation with a two-tailed test, and 95% confidence interval bands are depicted. Significances are depicted as *p < 0.05; ** p < 0.01. Scale bars represent 400 µm. HE, hematoxylin eosin; LFB, Luxol fast blue; Iba1, ionized calcium-binding adapter molecule 1; CD3, cluster of differentiation 3; FTY, fingolimod; EAE, experimental autoimmune encephalomyelitis.

To confirm the reduced clinical EAE course histologically, we investigated cell infiltration, demyelination and Iba1^+^ microglia/macrophages as well as CD3^+^ T cells in thoracic spinal cord sections. Histological examination of mice from the first experiment with the therapeutic treatment approach was performed (n=5; **Figure 4A**). The tissues were collected on day 58 after immunization (**Figure 4A**). Both FTY treatment and AFC-5128 at a dosage of 200 mg/kg body weight led to a significant decrease in cell infiltration in the thoracic spinal cord sections of chronic EAE mice (p<0.01; **Figure 4E**). The higher dosage of 400 mg/kg AFC-5128 was also able to reduce cell infiltration, but this effect was not significant (p=0.0508; **Figure 4E**). Higher levels of infiltration correlated with disease severity, as assessed via the sum of EAE scores (Spearman r=0.7572, p<0.0001; **Figure 4F**). Furthermore, demyelination was significantly reduced, with the most substantial effects observed using FTY (p<0.01; **Figure 4G**) and the higher dosages of 200 and 400 mg/kg AFC-5128 (p<0.01, p<0.05; **Figure 4G**). Similar to infiltration, the extent of demyelination and the total sum of EAE scores, as a measurement of disease severity, were correlated (Spearman r=0.6768, p=0.0002; **Figure 4H**). Additionally, there was a correlation between cell infiltration and demyelination of white matter (Spearman r=0.8254, p<0.0001; **Figure 4I**), indicating a valid evaluation and clinical correlation. A representative evaluation of subsets of the experimental groups (n=2-4 per group) revealed a trend toward lower numbers of Iba1^+^ and CD3^+^ cells in the white matter of the AFC-5128- and FTY-treated mice (**Figures 4J, K**), with a significant reduction in the number of Iba1^+^ cells in the group treated with FTY (p<0.05; **Figure 4J**).

### Therapeutic treatment with AFC-5128 could modify microglial marker expression

**Figure 5:**
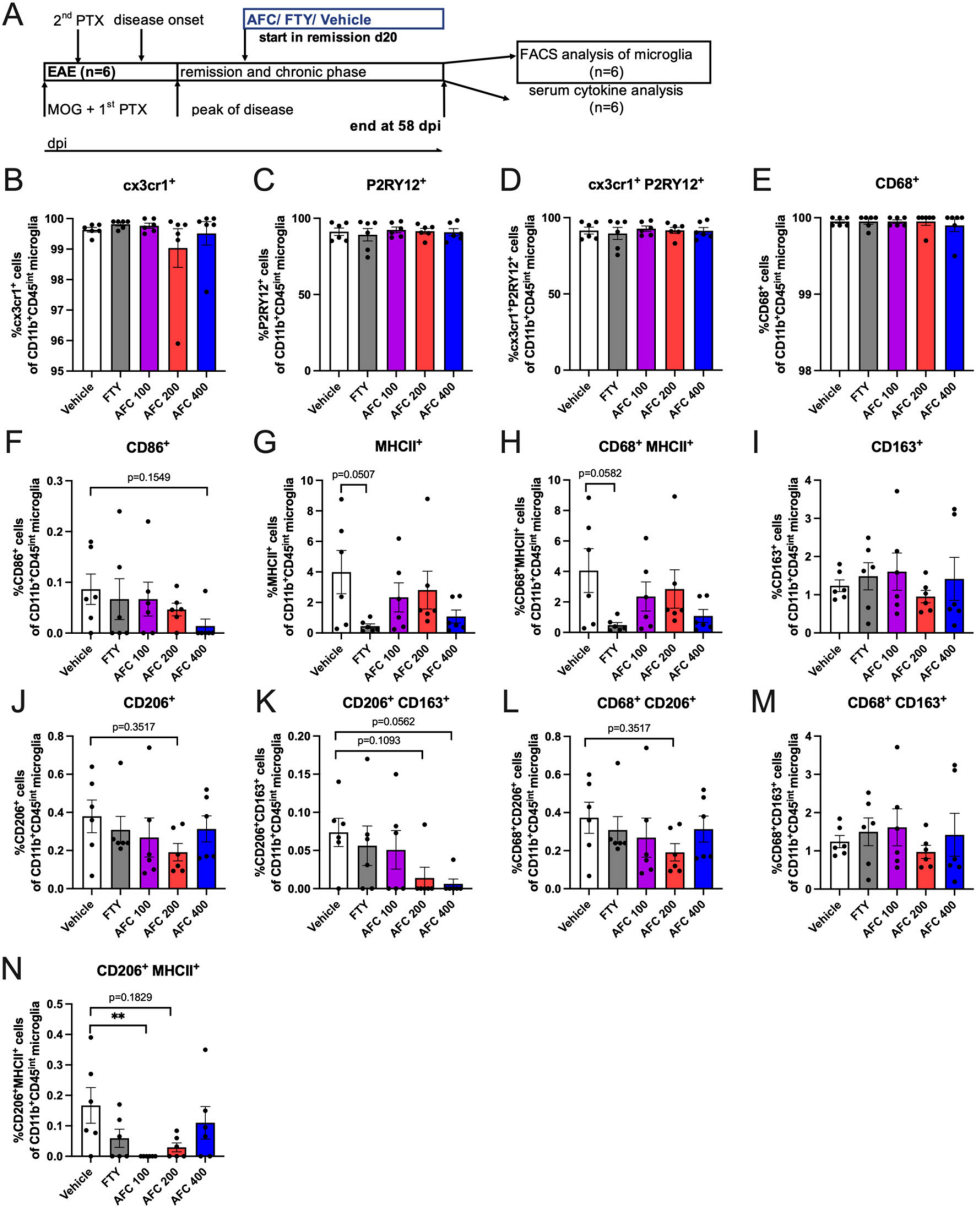
Marker expression of microglia in chronic EAE mice treated in a therapeutic paradigm. Single- and multiple-marker expression analysis. Brain-derived microglia from mice of the 2^nd^ experiment with therapeutic treatment (n=6), **(A)** were harvested at day 58 post EAE initiation and were analyzed via flow cytometry. Mice were treated from day 20 until day 58 post immunization **(A)**. The gating strategy is shown in **Supplementary Figure S1**. In terms of homeostasis markers, cx3cr1^+^ cells and P2RY12^+^ cells did not differ **(B, C, D)**. While AFC-5128 had no substantial effect on inflammation-associated markers **(F-H)**, FTY seemed to reduce the frequencies of MHCII^+^ cells and CD68^+^MHCII^+^ microglia **(G, H, E)** Single expression analysis revealed that CD68 was not altered. Although single-marker analysis revealed that CD163 was not affected in AFC-5128-treated mice, there was a trend toward decreased expression of phagocytosis-associated markers, as shown by the frequencies of CD206^+^ cells, CD206^+^ CD163^+^ cells and CD68^+^CD206^+^ cells **(I, J, K, L)**. FTY treatment did not alter the expression of markers associated with phagocytosis. **(M)** CD68^+^CD163^+^ cell frequencies did not change. **(N)** CD206^+^MHCII^+^ cell frequencies tended to decrease, reaching significance in the 100 mg/kg AFC-5128 group. The data are shown as the means ± SEMs. The data were analyzed via the Kruskal–Wallis test with *post hoc* Dunn’s multiple comparisons test. Significances are depicted as **p < 0.01. EAE, experimental autoimmune encephalomyelitis; FTY, fingolimod; cx3cr1, cx3c motif chemokine receptor 1; P2RY12, purinergic receptor P2Y12; CD86, cluster of differentiation 86; MHCII, major histocompatibility complex II; CD68, cluster of differentiation 68; CD163, cluster of differentiation 163; CD206, cluster of differentiation 206.

The phenotype of brain-derived microglia harvested at day 58 from chronic EAE mice treated in a therapeutic paradigm (2^nd^ exp., n=6; **Figure 5A** was analyzed through single- and multiple-marker expression analyses via flow cytometry. The mice were treated from day 20 up to day 58 post immunization (**Figure 5A**). In terms of homeostasis markers, the frequencies of cx3cr1^+^ cells and P2RY12^+^ cells did not significantly differ between the groups (**Figures 5B-D**). While AFC-5128 had no substantial effect on inflammation-associated markers (**Figures 5F-H**), FTY treatment particularly reduced the frequencies of MHCII^+^ cells and CD68^+^MHCII^+^ microglia (p=0.0507, p=0.0582; **Figures 5G, H**). However, the expression analysis of CD68 revealed no significant change under either FTY or AFC-5128 treatment compared with the control (**Figure 5E**). While the analysis of CD163 did not reveal significant changes in AFC-5128-treated mice, there was a noticeable trend toward lower expression of phagocytosis-associated markers. This was reflected in the frequencies of CD206^+^ cells, CD206^+^CD163^+^ cells, and CD68^+^CD206^+^ cells (**Figures 5I-L**). FTY treatment did not alter the expression of markers associated with phagocytosis (**Figure 5I-L**). The frequencies of CD68^+^CD163^+^ cells did not significantly change under the different treatments (**Figure 5M**). However, there was a trend toward lower frequencies of CD206^+^MHCII^+^ cells under AFC-5128 treatment (**Figure 5N**), reaching statistical significance in the group treated with 100 mg/kg body weight of AFC-5128 (p<0.01; **Figure 5N**). Analysis of absolute cell counts showed similar results (**Supplementary Figure S2**).

In summary, therapeutic treatment with AFC-5128 resulted in a trend to decreased expression of phagocytosis-associated markers on microglia while maintaining steady-state marker levels. FTY was able to reduce inflammation-associated markers on microglia.

### Therapeutic treatment with AFC-5128 in chronic EAE has no effect on peripheral immune cells or serum cytokine levels

**Figure 6:**
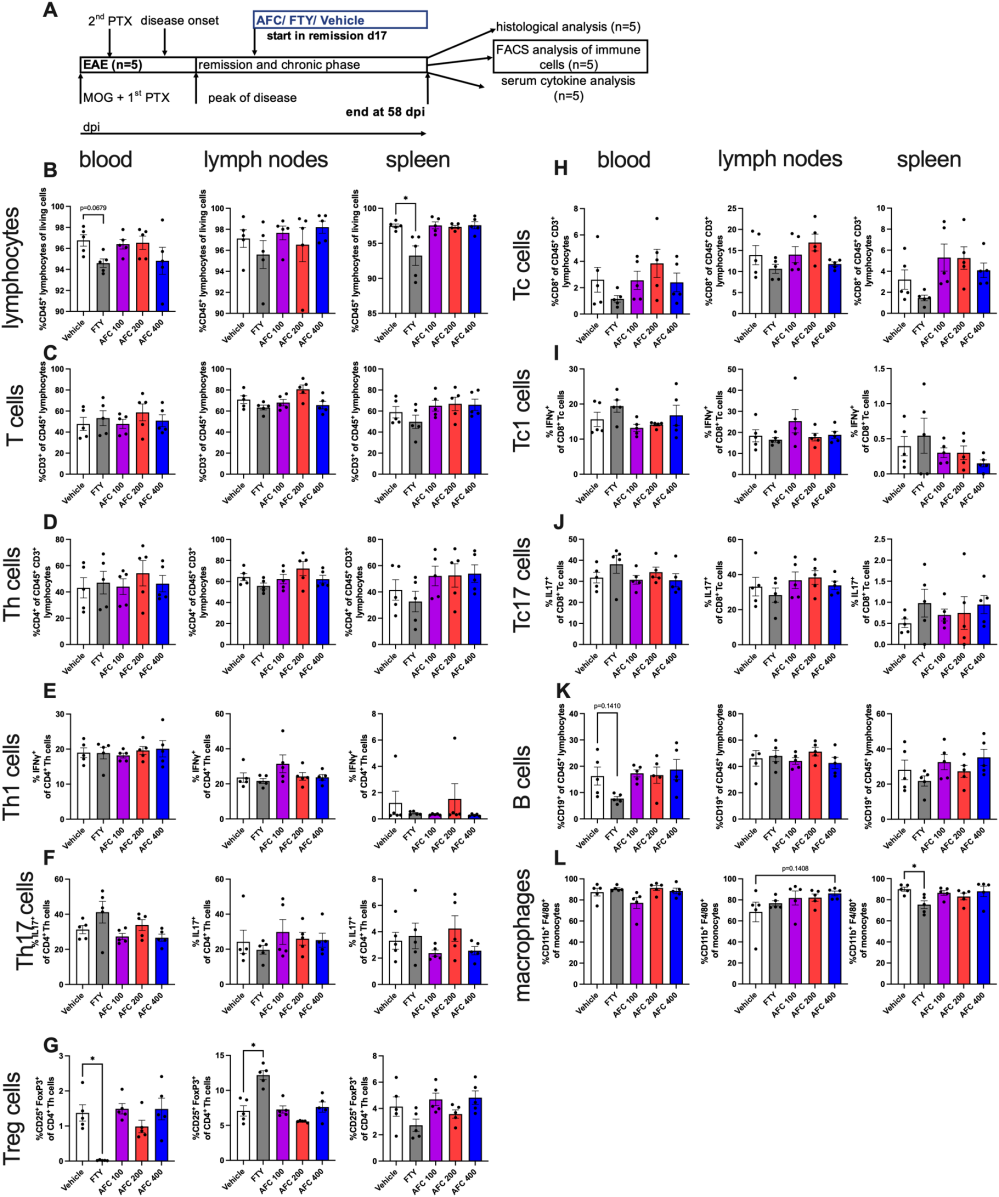
Immune cells from blood, lymph nodes and spleen from therapeutically treated chronic EAE mice were analyzed via flow cytometry. Immune cells from mice of the 1^st^ experiment with therapeutic treatment (n=5, **(A)**) were isolated at day 58 post EAE initiation and were analyzed via flow cytometry. Mice were treated from day 17 until day 58 postimmunization **(A)**. The cells were gated to exclude duplets, debris and dead cells. T cell subpopulations were related to parent. **(B)** Frequencies of lymphocytes in spleen were significantly reduced in the group with FTY treatment. **(C)** T cells did not differ. Even though frequencies of **(D)** Th cells, **(E)** Th1 cells and **(F)** Th17 cells were not altered with different therapies, **(G)** Treg cells were significantly reduced in blood and increased in lymph nodes in FTY-treated mice than in vehicle-treated mice. Frequencies of **(H)** Tc cells, **(I)** Tc1 cells and **(J)** Tc17 cells did not differ. **(K)** B cell frequencies in blood tended lower with FTY treatment. **(L)** The number of macrophages in spleen was significantly reduced in the FTY-treated group. The data are shown as the means ± SEMs. The data were analyzed using the Kruskal–Wallis test with *post hoc* Dunn’s multiple comparisons analysis. Significances are depicted as *p < 0.05. FTY, fingolimod; Th cells, T helper cells; Tc cells, cytotoxic T cells; Treg cells, regulatory T cells.

**Figure 7:**
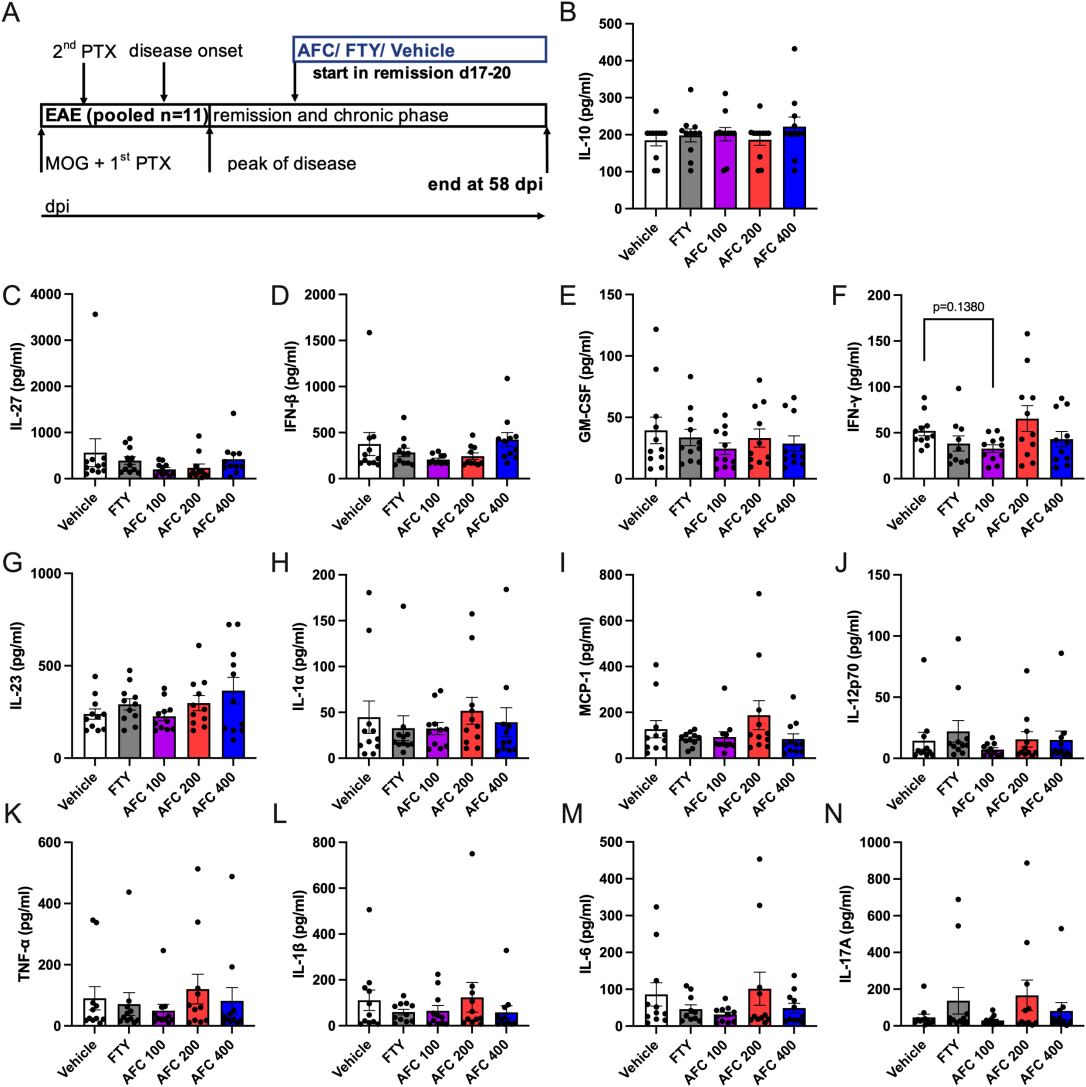
Cytokines of serum supernatants from chronic EAE mice were not affected following therapeutic treatment. Serum supernatants from mice of both experiments with therapeutic treatment (1^st^ ex. n=5, 2^nd^ exp. n=6, total n=11) were collected at day 58 post EAE initiation **(A)**. The mice were treated from days 17- -20 until day 58 postimmunization **(A)**. The serum supernatants were analyzed via the Legendplex^®^ cytokine bead assay. Overall, AFC and FTY did not modulate secretion of cytokines in the therapeutic treatment approach with chronic EAE progression. AFC tended to reduce the concentration of F) IFNγ. All the analyses were performed in duplicates. The data are shown as the means ± SEMs. The data were analyzed via the Kruskal–Wallis test with *post hoc* Dunn’s multiple comparisons test. EAE, experimental autoimmune encephalomyelitis; FTY, fingolimod; IFNγ, Interferon gamma.

To understand the potential effect of AFC-5128 on peripheral immune cells and serum cytokines, we investigated immune cell alterations in the blood, spleen and lymph nodes via flow cytometry and analyzed the serum supernatants via a Legendplex^®^ cytokine bead assay. Immune cells from chronic EAE mice of the 1^st^ experiment (n=5, **Figure 6A**) and serum supernatants of both experiments with the therapeutic treatment paradigm (**Figure 7A**) were obtained on day 58 post immunization. Mice were treated from day 17/20 up to day 58 post EAE immunization as indicated in respective figures (**Figures 6A**, **7A**).

The frequency of CD45^+^ lymphocytes in the spleen was reduced in FTY-treated mice (p<0.05, **Figure 6B**). CD3^+^ T cells did not differ under different treatments (**Figure 6C**). CD4^+^ Th cells, Th1 (CD4^+^IFNγ^+^) cells and Th17 (CD4^+^IL17^+^) cells were not considerably affected (**Figures 6D-F**). As expected by the S1PR1 antagonism, the frequency of regulatory T cells (CD25^+^FoxP3^+^) was significantly reduced in the blood (p<0.05) but increased in the lymph nodes (p<0.05, **Figure 6G**) in FTY-treated mice than in vehicle-treated mice. However, the frequencies of CD8^+^ Tc cells, Tc1 (CD8^+^IFNγ^+^) cells and Tc17 (CD8^+^IL17^+^) cells were not altered under either AFC-5128 or FTY therapy (**Figures 6H-J**). The frequency of B cells in the blood slightly decreased in FTY-treated mice, but this difference was not significant (p=0.1410, **Figure 6K**). The frequency of macrophages in the spleen was reduced in FTY-treated mice (p<0.05, **Figure 6L**). AFC-5128 had no substantial effects on B cells or macrophages. Analysis of absolute cell counts showed similar results (**Supplementary Figure S3**).

Most serum cytokine levels were not significantly altered in either the AFC-5128- or the FTY-treated mice (**Figure 7**). AFC-5128 tended to reduce IFNγ levels in the 100 mg/kg AFC-5128 group (p=0.1380, **Figure 7F**).

In summary, FTY retains specific immune cell populations in the secondary lymphatic organs of mice with chronic EAE via the expected S1PR1 antagonism, whereas AFC-5128 seems to have no substantial effects on peripheral immune cells or serum cytokines in the experimental approach presented here (analysis at a very late stage of the disease).

### Late therapeutic treatment with AFC-5128 has mild effects on chronic EAE

**Figure 8:**
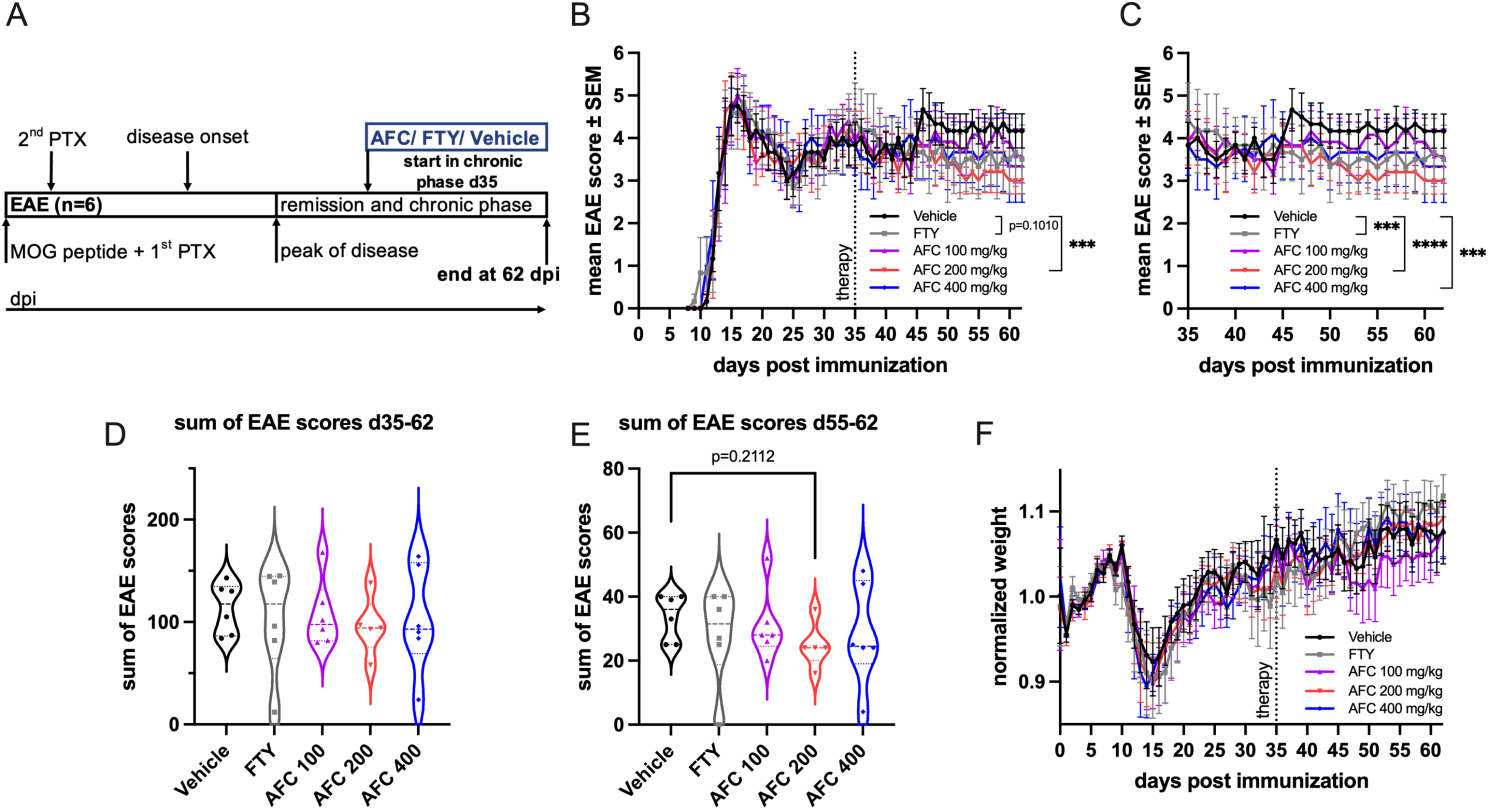
Late therapeutic AFC-5128 treatment has mild effects on chronic MOG-EAE. **(A)** 8- to 10-week-old C57BL/6 mice were immunized with s.c. MOG_35-55_ injection and treated i.p. with PTX on days 0 and 2 post immunization. The mice were treated 2x/d with vehicle, 1 mg/kg body weight FTY per day or different dosages of AFC-5128 by oral gavage from day 35 (early chronic phase) to day 62 postimmunization. **(B)** Treatment with 200 mg/kg body weight AFC-5128 significantly reduced overall EAE scores. Overall EAE scores tended to be lower when FTY was used. **(C)** Upon analysis of the period of treatment (d35-d62), both FTY and AFC-5128 at dosages of 200 and 400 mg/kg significantly reduced EAE scores. The effects were not reflected in **(D)** the sum of EAE scores of the treatment period and **(E)** the normalized weight. n=6 per group. The data are shown as the means ± SEMs. The weight was normalized to the respective group mean. The data were analyzed via the Kruskal–Wallis test with *post hoc* Dunn’s multiple comparisons analysis. Significances are depicted as ***p < 0.001; ****p < 0.0001. EAE, experimental autoimmune encephalomyelitis; MOG, Myelin oligodendrocyte glycoprotein; s.c., subcutaneously; i.p., intraperitoneally; PTX, pertussis toxin; FTY, fingolimod.

After showing that AFC-5128 is effective in both preventive and therapeutic treatment paradigms, we wanted to investigate AFC-5128 in a late-treatment approach for chronic EAE. Therefore, we performed an experiment with the start of treatment beginning on day 35 (**Figure 8A**). The dosage of 200 mg/kg AFC-5128 led to an overall reduction of EAE scores (p<0.001, **Figure 8B**). FTY did not significantly reduce overall EAE scores (p=0.1010). When only the period of treatment was considered (d35-d62, **Figure 8C**), both AFC-5128 at higher doses (200 mg/kg p<0.0001; 400 mg/kg p<0.001) and FTY (p<0.001) led to significantly reduced overall EAE scores. However, it should be noted that the effects were not reflected in the sum of EAE scores of the treatment period (**Figure 8D**), nor in reduced weight loss (**Figure 8F**). When only the sum of EAE scores at the very late period d55-62 was considered, the treatment with 200 mg/kg AFC-5128 tended to reduce the disease severity (p=0.2112; **Figure 8F**).

### AFC-5128 treatment *in vivo* reduces *ex vivo* T-cell proliferation upon MOG-restimulation

**Figure 9:**
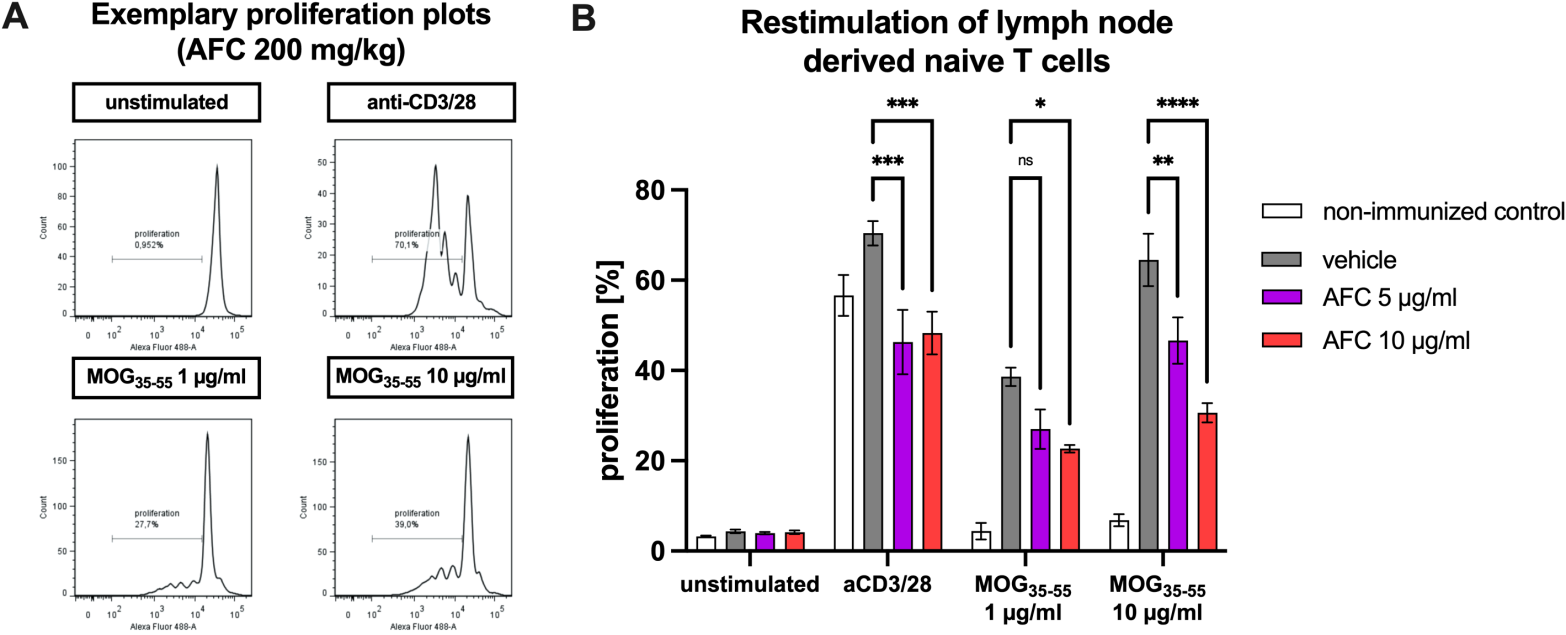
T-cell proliferation upon antigen restimulation. Female C57BL/6 mice were immunized with MOG_(35-55)_ and treated with AFC-5128 or vehicle or left untreated (control). After 10 days, the lymph node-derived naïve T cells were isolated and incubated with vehicle control, 5 µg/ml AFC-5128 (bioequivalent to 100 mg/kg) or 10 µg/ml AFC-5128 (bioequivalent to 200 mg/kg). After preconditioning, the T cells were labeled with CFSE. In parallel, BMDCs were isolated and differentiated into dendritic cells. BMDCs were then restimulated with 1 or 10 µg/ml MOG_(35-55)_ peptide for 24 h to achieve effective antigen processing. Preconditioned T cells (2.25x10^6^) were incubated with 2.5x10^5^ BMDCs. Anti-CD3/28 antibody stimulation served as a positive control, whereas unstimulated condition was used as a negative control. After 4 days of incubation, T-cell proliferation was analyzed via flow cytometry. **(A)** Exemplary proliferation plots and **(B)** the frequency of proliferating cells of stimulated T cells obtained from mice treated with 200 mg/kg AFC-5128 are shown. For statistical analysis, two-way ANOVA, followed by Tukey’s multiple comparisons test was used to identify differences compared with the vehicle group. The data are shown as the means ± SEMs of 2 individual experiments with 3–4 mice per group each. The nonimmunized control condition was examined in one experiment with 4 mice. Significances are depicted as *p < 0.05; **p < 0.01; ****p < 0.0001. EAE, experimental autoimmune encephalomyelitis; MOG, myelin oligodendrocyte glycoprotein; CD3, cluster of differentiation 3; CD28, cluster of differentiation 28; BMDCs, bone marrow-derived cells; CFSE, carboxyfluorescein succinimidyl ester.

To assess whether AFC-5128 might also influence the proliferation of lymph node-derived T cells, T cells were isolated from EAE mice treated with vehicle or 100 mg/kg or 200 mg/kg AFC-5128. Stimulation of vehicle-treated EAE mice with aCD3/aCD28 led to marked T-cell proliferation of 70.4%. AFC-5128 treatment *in vivo* reduced *ex vivo* T-cell proliferation upon MOG-restimulation at both dosages (p<0.001; **Figures 9A, B**). Restimulation with 1 µg/ml MOG_35-55_ and 10 µg/ml MOG_35-55_ also increased T-cell proliferation, which, more importantly, was significantly reduced after *in vivo* treatment with AFC-5128 and *ex vivo* restimulation with MOG_35-55_ (stimulation with 10 µg/ml MOG_35-55_, treatment with AFC-5128 100 mg/kg p<0.01; 200 mg/kg p<0.0001). Notably, the inhibitory effect of AFC-5128 on MOG restimulation was even more pronounced than that on nonspecific aCD3/aCD28 restimulation.

## Discussion

Despite major advances made in the treatment of multiple sclerosis in recent decades, there are still situations where even highly effective immunotherapies have limited efficacy, particularly in the progressive phase of the disease (2, 5). Modulation of microglia has been discussed as a potential therapeutic avenue to tackle these cells as a main driver of progression.

In this study, we investigated the effects of the P2X7R-inhibiting molecule AFC-5128 on microglial calcium homeostasis and its therapeutic potential in both acute and chronic experimental autoimmune encephalomyelitis (EAE). *In vitro*, AFC-5128 significantly reduced ATP-mediated calcium uptake in primary murine microglia, suggesting effective modulation of P2X7R activity. *In vivo*, AFC-5128 improved the course of acute EAE, as well as disability progression in chronic EAE in a therapeutic treatment paradigm. Notably, FTY, a known strong modulator of EAE (29), attenuated clinical signs of acute and chronic EAE as well. Both therapeutic AFC-5128 and FTY treatment led to a significant reduction in cell infiltration and demyelination in the spinal cord of chronic EAE mice, both of which strongly correlated with clinical scores, suggesting that these treatments effectively diminished the inflammatory response within the spinal cord of EAE mice. AFC-5128 tended to reduce the expression of phagocytosis-associated markers in the brain-derived microglia of chronic EAE mice. However, peripheral immune cells and serum cytokines remained unchanged in our experimental setup.

The question arises as to the mechanisms through which AFC-5128 caused these effects. Other P2X7R antagonists, such as oxATP or Brilliant Blue G (BBG), have also shown remarkable effects on neuroinflammation (16, 30–33). BBG reduces neurological symptoms, demyelination, and inflammatory cell infiltration and improves nerve conditions in rats with experimental autoimmune neuritis (EAN) (31). Moreover, BBG decreases the production of proinflammatory cytokines and reduces the expression of P2X7R and the associated NLRP3 inflammasome. Furthermore, it suppresses the differentiation of Th1 and Th17 cells, thereby protecting against EAN (31). Both Th1 and Th17 cells represent important sources of inflammatory cytokines during CNS inflammation, including EAE (34). These findings suggest that AFC-5128 could similarly attenuate EAE, although we were unable to demonstrate this when peripheral immune cells were examined, which could be due to the long experimental duration and the analysis at such a late stage in disease.

Notably, mesenteric T cells of EAE mice express P2X7R and primarily infiltrate the white matter (35). This suggests that orally available therapies might be particularly effective. As we demonstrated in our work, the oral administration of AFC-5128 and FTY was effective, especially in the therapeutic treatment paradigm, suggesting effects on this mesenteric T-cell compartment. This finding is supported by the immunohistochemistry results from the therapeutic treatment experiment, which revealed reduced infiltrating T-cell numbers in the white matter of AFC-5128- and FTY-treated mice, as well as the inhibition of T-cell proliferation upon antigen stimulation. Other studies have shown that the receptor is an important mediator of lymphocytic homeostasis (36). In conclusion, although we could not demonstrate an effect of AFC-5128 on immune cells, a possible beneficial effect in this respect cannot be excluded. Further studies, especially in the early stages of EAE, are needed to evaluate the effect of AFC-5128 on immune cells with certainty.

Microglia play important roles in the pathogenesis and maintenance of neuroinflammation in chronic progressive forms of MS and therefore represent important therapeutic targets (2, 6, 37, 38). Microglia constantly scan the environment and can adopt a spectrum of different states between inflammatory and protective states, which is why modulation seems promising (38). Elevated numbers of P2X7R-immunoreactive microglia were found in the postmortem spinal cords of MS and ALS patients (15). P2X7R promotes the modulation and proliferation of microglia (9, 12). In EAE, changes in microglia are recognized before the first clinical signs appear, and these changes are associated with increased expression of P2X7R (17, 33). We demonstrated that AFC-5128 could possibly modulate the expression of markers of microglia, as seen in a trend toward reduced expression of phagocytosis-associated markers. Moreover, the number of Iba1^+^ microglia/macrophages tends to decrease in spinal cord sections, which is in line with other findings of the modulation of microglia by P2X7R antagonism (33). While inflammation-associated microglia mainly appear in active lesions at the early stages of MS, phagocytosis-associated markers such as CD163 and CD206 peak in late active MS lesions (17, 37), the downregulation mediated by AFC-5128 could thus be due to a reduced number of lesions, which in turn corresponds to improved clinical conditions. In our study, reduced demyelination and infiltration were strongly correlated with lower EAE scores. However, we did not detect any differences in the expression of the homeostasis markers cx3cr1^+^ and P2RY12^+^. Possible reasons for the overall suggestive but barely significant effects of microglial markers are the small group size and the long-term clinical course in our study. Over the course of the study, the clinical condition of most of the animals hardly changed, so we assume that the disease activity and associated cellular changes were reduced and slowed overall. Future experiments analyzing mouse microglia should be performed earlier in the EAE to strengthen the evidence of possible effects.

Currently, the role of calcium in MS is attracting increasing interest (39). Under pathological conditions, excitotoxicity occurs, which affects the calcium homeostasis of various cell types, including glia. This leads to calcium shifts via ion channels, which strengthens the processes and *vice versa* (40–42). By showing *in vitro* that AFC-5128 significantly reduces microglial calcium influx, we provide evidence that AFC-5128 effectively modulates the P2X7R and is possibly neuroprotective against excitotoxicity in MS.

Regarding the clinical effect of AFC-5128, it is striking that the medium dosage of 200 mg/kg is particularly effective. Since the higher dose of 400 mg/kg AFC-5128 did not seem to be more effective, we suspect a ceiling effect. Different studies have shown that the expression of P2X7R is highest at the peak and in remission of EAE (43, 44). Treatment with AFC-5128 from the chronic phase onward had only mild effects, but notably, AFC-5128 was still able to positively influence EAE at such a late stage. Together, these findings indicate that there is not only an optimal dose range but also a therapeutic time window for treatment with AFC-5128. However, it also has the potential to be effective in the late phases of the disease.

In light of the different results from other studies, the extent to which AFC-5128 also influences other cell types remains unknown. While P2X7R expression is reduced in PBMCs in the acute phase in RRMS patients and EAE rodents, P2X7R expression is upregulated in astrocytes in the frontal cortex of SPMS patients (11). Further research is needed to understand the potential of P2X7R blockade and its impact on other cell types.

Next, it is important to discuss the differences between FTY and AFC-5128. As an S1PR modulator, FTY remains an effective therapy for the acute forms of MS. FTY retains reactive immune cells in the secondary lymphatic organs, leading to significantly reduced infiltration of immune cells into the CNS, particularly in EAE. Progressive forms of MS are characterized by CNS-intrinsic inflammation, which we are targeting with P2X7R blockade, and furthermore expect effects on microglia. Thus, AFC-5128 is probably suitable for progressive forms of MS, whereas FTY was proven to be not effective (45).

There are some limitations that need to be addressed. To date, AFC-5128 has been used only *in vivo* in studies of selective seizure models (46, 47). First, there is no optimal model that reflects all the characteristics of chronic multiple sclerosis, including the EAE model used here (48). While there were strong and reliable effects in the preventive and therapeutic treatment paradigms, the late therapeutic treatment with AFC-5128 from the chronic phase onward had only mild effects on the course of chronic EAE. This might also be because the chronic EAE paradigm does not perfectly reflect the situation in long-lasting MS. However, despite of the revealed potential effect of AFC-5128 in the preventive and late therapeutic treatment paradigm, further investigation of possible cellular changes by FACS analysis or histology should be performed in future experiments.

## Conclusion

In conclusion, we have provided evidence that treatment with AFC-5128 ameliorates acute and chronic EAE, with beneficial effects on histological markers such as white matter infiltration and demyelination. AFC-5128 reduces T-cell proliferation upon antigen stimulation, may modulate microglial marker expression *in vivo* and reduces microglial calcium influx *in vitro*, highlighting its potential as a promising therapeutic candidate for relapsing-remitting and chronic progressive MS. Further studies are needed to investigate the effects of AFC-5128 on additional cell types and to assess its safety, efficacy, and potential adverse effects in the clinical setting.

## Data Availability

The raw data supporting the conclusions of this article will be made available by the authors, without undue reservation.
