# Correction: The P2X7R-antagonist AFC-5128 ameliorates chronic experimental autoimmune encephalomyelitis in a preventive and therapeutic paradigm

**DOI:** 10.3389/fimmu.2025.1652299

**Published:** 2025-07-24

**Authors:** Robert Hoffrogge, Anna Karachunskaya, Neele Heitmann, Steffen Haupeltshofer, Xiomara Pedreiturria, Katharina Klöster, Verian Bader, Konstanze F. Winklhofer, Michael Hamacher, Bert Klebl, Ralf Gold, Klaus Dinkel, Ingo Kleiter, Simon Faissner

**Affiliations:** ^1^ Department of Neurology, St. Josef-Hospital, Bochum, Germany; ^2^ Translational Neurology Group, Department of Clinical Science, Lund University, Lund, Sweden; ^3^ Molecular Cell Biology, Ruhr-University, Bochum, Germany; ^4^ Affectis Pharmaceuticals AG, Dortmund, Germany; ^5^ KHAN Technology Transfer Fund I GmbH & Co KG, Dortmund, Germany; ^6^ Lead Discovery Center GmbH, Dortmund, Germany; ^7^ Behandlungszentrum Kempfenhausen für Multiple Sklerose Kranke gemeinnützige GmbH, Berg, Germany

**Keywords:** P2X7R, progressive multiple sclerosis, microglia, neuroinflammation, neuroprotection

Author “Steffen Haupeltshofer” was omitted as an author in the published article. The correct author list reads:

“Robert Hoffrogge^1^, Anna Karachunskaya^1^, Neele Heitmann^1^, Steffen Haupeltshofer^2^, Xiomara Pedreiturria^1^, Katharina Klöster^1^, Verian Bader^3^, Konstanze F. Winklhofer^3^, Michael Hamacher^4^, Bert Klebl^5^, Ralf Gold^1^, Klaus Dinkel^6^, Ingo Kleiter^1,7^, Simon Faissner^1*^

^1^Department of Neurology, St. Josef-Hospital, Bochum, Germany

^2^Translational Neurology Group, Department of Clinical Science, Lund University, Lund, Sweden

^3^Molecular Cell Biology, Ruhr-University, Bochum, Germany

^4^Affectis Pharmaceuticals AG, Dortmund, Germany

^5^KHAN Technology Transfer Fund I GmbH & Co KG, Dortmund, Germany

^6^Lead Discovery Center GmbH, Dortmund, Germany

^7^Behandlungszentrum Kempfenhausen für Multiple Sklerose Kranke gemeinnützige GmbH, Berg, Germany”

The **Author contributions statement** has been corrected to read:

“RH: Writing – original draft, Writing – review & editing, Conceptualization, Data curation, Formal Analysis, Investigation, Methodology, Visualization. AK: Writing – review & editing, Formal Analysis, Investigation, Methodology, Visualization. NH: Writing – review & editing, Conceptualization, Data curation, Formal Analysis, Investigation, Methodology, Validation, Visualization. SH: Writing – original draft, Writing – review & editing, Conceptualization, Data curation, Formal Analysis, Investigation, Methodology, Validation, Visualization.XP: Writing – review & editing, Formal Analysis, Investigation, Methodology, Project administration, Resources, Validation. KK: Writing – review & editing, Data curation, Formal Analysis, Investigation, Methodology. VB: Writing – review & editing, Formal Analysis, Investigation, Methodology, Software, Visualization. KW: Writing – review & editing, Formal Analysis, Methodology, Resources, Software. MH: Writing – review & editing, Resources. BK: Writing – review & editing, Resources. RG: Writing – review & editing, Funding acquisition, Resources. KD: Writing – review & editing, Resources. IK: Writing – review & editing, Conceptualization, Formal Analysis, Funding acquisition, Supervision, Visualization. SF: Writing – original draft, Writing – review & editing, Conceptualization, Data curation, Formal Analysis, Funding acquisition, Methodology, Project administration, Resources, Supervision, Validation, Visualization.”

The original version of this article has been updated.

