# Supplementary material for: The P2X7R-antagonist AFC-5128 ameliorates chronic experimental autoimmune encephalomyelitis in a preventive and therapeutic paradigm
Source: Front Immunol. 2025 Apr 16;16:1554999. doi: 10.3389/fimmu.2025.1554999 (PMC12040686; doi:10.3389/fimmu.2025.1554999)
Supplement: Supplementary file 1 [file DataSheet1.docx]

**The P2X7R-antagonist AFC-5128 ameliorates chronic experimental autoimmune encephalomyelitis in a preventive and therapeutic paradigm**

Robert Hoffrogge^1^, Anna Karachunskaya^1^, Neele Heitmann^1^, Xiomara Pedreiturria^1^, Katharina Klöster^1^, Verian Bader^2^, Konstanze F. Winklhofer^2^, Michael Hamacher^3^, Bert Klebl^4^, Ralf Gold^1^, Klaus Dinkel^5^, Ingo Kleiter^1,6^, Simon Faissner^1^

^1^ Department of Neurology, Ruhr-University Bochum, St. Josef-Hospital, Bochum, Germany

^2^ Molecular Cell biology, Ruhr-University Bochum, Germany

^3^ Affectis Pharmaceuticals AG

^4^ KHAN Technology Transfer Fund I GmbH & Co KG

^5^ Lead Discovery Center GmbH

^6^ Behandlungszentrum Kempfenhausen für Multiple Sklerose Kranke gemeinnützige Gmbh, 82335 Berg, Germany

Corresponding author:

Prof. Dr. Simon Faissner, MD

Department of Neurology

Ruhr-University Bochum, St. Josef-Hospital

Gudrunstr. 56, 44791 Bochum, Germany

Tel: +49-234-5092411; Fax: +49-234-5092740

Email: simon.faissner@rub.de

**SUPPLEMENT**

**Supplementary figures**


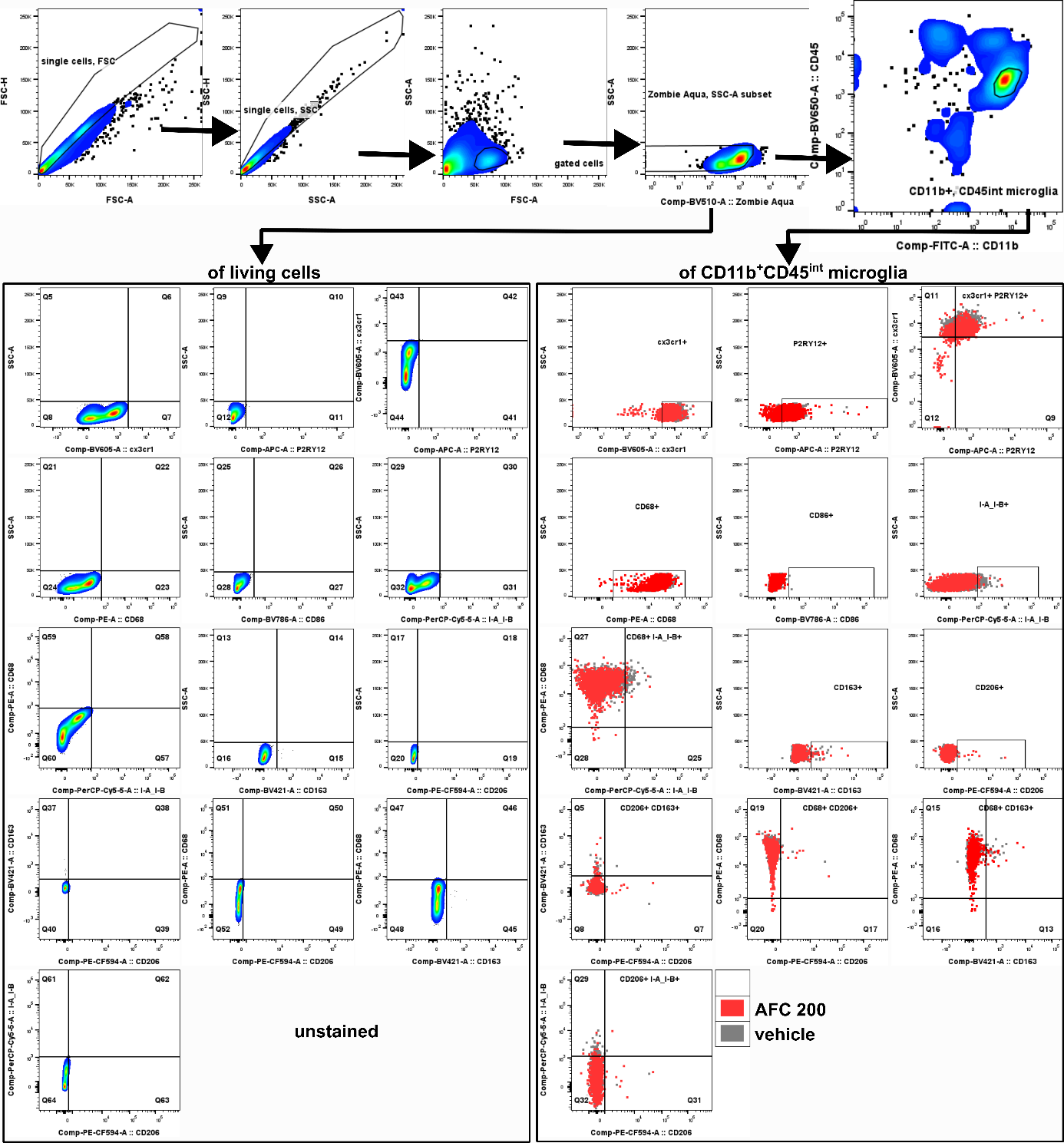


**Figure S1: gating strategy for brain derived microglia flow cytometry with FlowJo.** Exemplary gating strategy for microglia isolated from brain tissue by Percoll gradient. The thresholds for positive stained cell populations was detected using unstained cells (left side). On the right side we show exemplary overlay graphs with each an untreated sample and a sample from a AFC 200 treated sample.


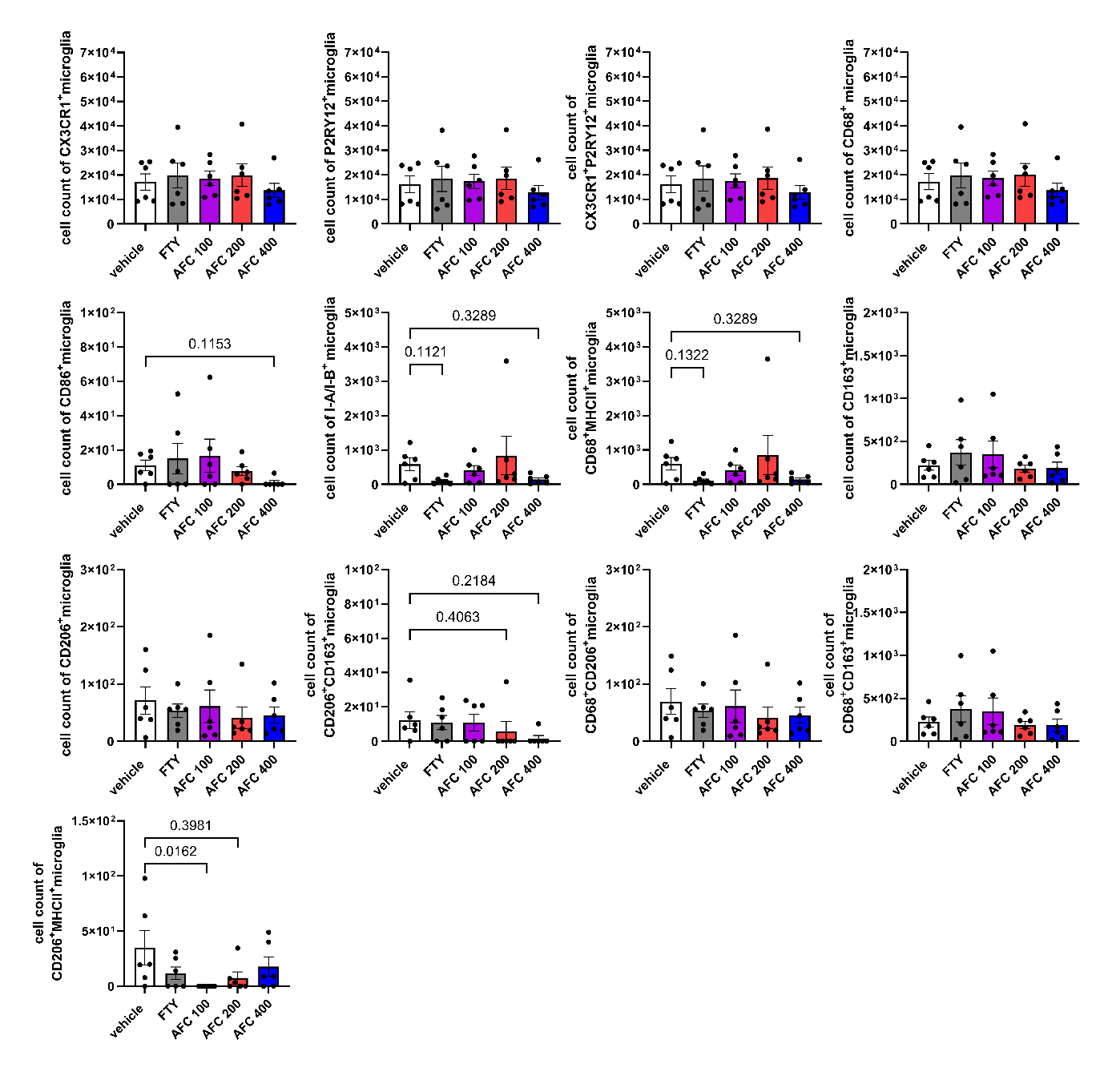


**Figure S2: Flow cytometry analysis with absolute cell counts corresponding to Figure 5.** Frequencies of living gated cells of the cell populations shown in Figure 5 were multiplied with the total amount of isolated cells for each mouse to calculate the shown cell count.


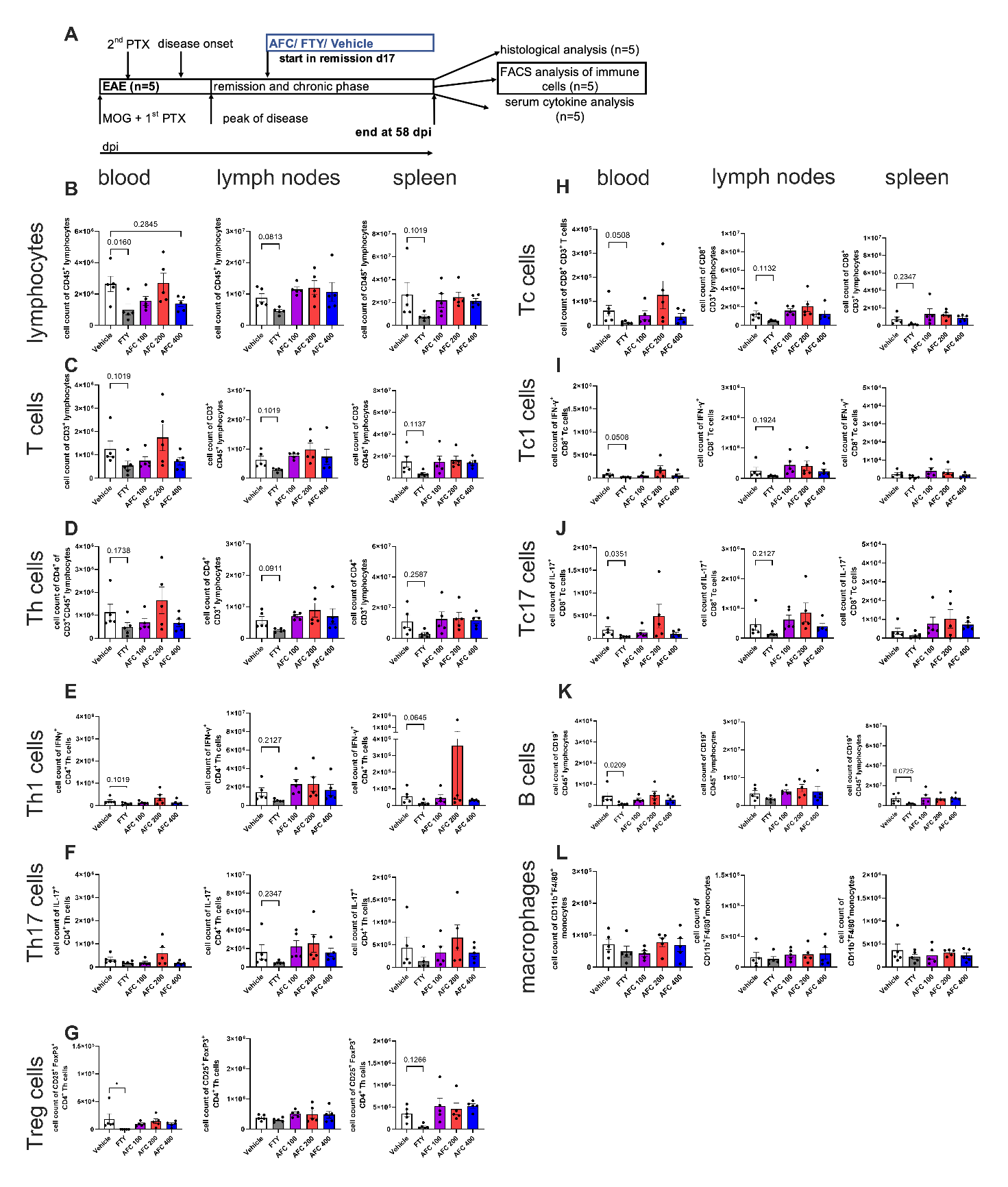


**Figure S3: Flow cytometry analysis with absolute cell counts corresponding to Figure 6.** Frequencies of living cells of the populations shown in Figure 6 were multiplied with the total amount of isolated cells of the blood, spleen or inguinal lymph nodes sample for each mouse to calculate the shown cell count.

**Supplementary tables**

**Table S1a: Medium used for microglia isolation**

| **Name** | **Concentration** | **Vendor** |
| --- | --- | --- |
| RPMI medium | - | gibco, REF: 31870-025 |
| Fetal Calf Serum (FCS) | 5% | PAN-Biotech REF: P30-3306 |
| Glutamine | 1% | Thermo Fisher Scientific REF: 25030024 |
| Penicillin/Streptomycin | 1% | Thermo Fisher Scientific REF: 15140122 |
| beta-mercaptoethanol | 0,1% | Sigma Aldrich REF: M3148 |

**Table S1b: Antibodies used for flow cytometry**

| **Antibody** | **Vendor** | **REF** | **Dilution** |
| --- | --- | --- | --- |
| Anti-mouse CD86 BV785 | BioLegend | 105043 | 1:200 |
| Anti-mouse CD11b FITC | BioLegend | 101205 | 1:200 |
| Anti-mouse CD45 BV650 | BioLegend | 103151 | 1:200 |
| Anti-mouse CX3CR1 BV605 | BioLegend | 149027 | 1:50 |
| Anti-mouse CD163 BV421 | BioLegend | 155309 | 1:400 |
| Anti-mouse MHCII PerCP/Cy5.5 | BioLegend | 107625 | 1:400 |
| Anti-mouse P2RY12 APC | BioLegend | 848005 | 1:200 |
| Anti-mouse CD68 PE | BioLegend | 137013 | 1:100 |
| Anti-mouse CD206 PE-Dazzle594 | BioLegend | 141731 | 1:50 |
| True-Stain Monocyte Blocker™ | BioLegend | 426103 | 1:20 |
| Anti-Mouse F4/80 BV421 | BioLegend | 123132 | 1:50 |
| Anti-Mouse CD3 AF700 | BioLegend | 100216 | 1:100 |
| Anti-Mouse CD4 BV785 | BioLegend | 100552 | 1:200 |
| Anti-Mouse CD8a PerCP/Cy5.5 | BioLegend | 100733 | 1:200 |
| Anti-Mouse CD19 APC-Cy7 | BD Biosciences | 557655 | 1:200 |
| Anti-Mouse CD25 APC | eBiosciences | 17-0251-81A | 1:200 |
| Anti-Mouse FoxP3 PE | eBiosciences | 12-5773-82 | 1:200 |
| Anti-Mouse IFNγ BV605 | BioLegend | 505839 | 1:100 |
| Anti-Mouse IL17a PE-Dazzle594 | BioLegend | 506937 | 1:50 |
| Anti-Mouse CD16/32 (Fc-Block) | BioLegend | 101320 | 1:200 |
| Zombie Aqua | BioLegend | 423101 | 1:1500 |

**Table S1c: Antibodies used for immunohistochemistry**

| **Antibody** | **Dilution** | **Species** | **REF** | **Vendor** |
| --- | --- | --- | --- | --- |
| anti-Iba-1 | 1:100 | rabbit | 019-19741 | WAKO Fujifilm |
| Alexa Fluor^®^ 488 anti-rabbit IgG | 1:1000 | goat | A32731 | Invitrogen |
| anti-CD3 | 1:200 | rat | MCA1477 | BIO-RAD |
| Alexa Fluor^®^ 555 anti-rat | 1:1000 | donkey | A48270 | Invitrogen |
| DAPI-Fluoromount | - | - | 0100-20 | Southern Biotech |

**Table S2a: Medium for primary microglia culture**

| **Name** | **Concentration** | **Vendor** |
| --- | --- | --- |
| DMEM/F12 GlutaMax^TM^ medium | - | Thermo Fisher Scientific REF: 31331093 |
| Fetal Calf Serum (FCS) | 10% | PAN-Biotech REF: P303306 |
| Penicillin/Streptomycin | 1% | Thermo Fisher Scientific REF: 15140122 |

**Table S2b: Medium for primary microglia experiments**

| **Name** | **Concentration** | **Vendor** |
| --- | --- | --- |
| Neurobasal™ medium | - | Gibco™ REF: 21103049 |
| B27 supplement | 2% | Life Technologies REF: 17504044 |
| Penicillin/Streptomycin | 1% | Thermo Fisher Scientific REF: 15140122 |
| GlutaMAX™ | 1% | Thermo Fisher Scientific REF: 35050038 |

**Table S2c: Fluo-4-loading-solution for Ca^2+^-imaging**

| **Name** | **Volume, total 1 ml** | **Vendor** |
| --- | --- | --- |
| 100x PowerLoad^TM^ Concentrate | 10µl | Thermo Fisher Scientific REF: P10020 |
| 1000x Fluo-4 AM | 1µl | Thermo Fisher Scientific REF: F14201 |
| 20mM glucose in PBS | 989µl | Thermo Fisher Scientific REF: 15140122 |

**Table S2d: Stimulation solution for Ca^2+^-imaging**

| **Name** | **Concentration** | **Vendor** |
| --- | --- | --- |
| HBSS w/o Ca^2+^ | - | Thermo Fisher Scientific REF:14170138 |
| ATP | 5 mM | Sigma Aldrich, REF: A6419-1G, solved in A. dest, pH 7,4 adjusted and sterile filtered |
| CaCl_2_ | 2,5 µM | Sigma Aldrich REF: C8106-500G, solved in A. dest, sterile filtered |
| AFC-5128 | 10 µM | Affectis GmbH, solved in 100% DMSO |
